# Failure of diet-induced transcriptional adaptations in alpha-synuclein transgenic mice

**DOI:** 10.1093/hmg/ddac205

**Published:** 2022-08-24

**Authors:** Alexander Kilzheimer, Thomas Hentrich, Carola Rotermund, Philipp J Kahle, Julia M Schulze-Hentrich

**Affiliations:** Institute of Medical Genetics and Applied Genomics, University of Tübingen, 72074 Tübingen, Germany; Institute of Medical Genetics and Applied Genomics, University of Tübingen, 72074 Tübingen, Germany; Laboratory of Functional Neurogenetics, Department of Neurodegeneration, Hertie Institute for Clinical Brain Research, University of Tübingen, 72074 Tübingen, Germany; German Center for Neurodegenerative Diseases (DZNE), 72074 Tübingen, Germany; Laboratory of Functional Neurogenetics, Department of Neurodegeneration, Hertie Institute for Clinical Brain Research, University of Tübingen, 72074 Tübingen, Germany; Institute of Medical Genetics and Applied Genomics, University of Tübingen, 72074 Tübingen, Germany; Institute for Bioinformatics and Medical Informatics (IBMI), University of Tübingen, 72074 Tübingen, Germany

## Abstract

Nutritional influences have been discussed as potential modulators of Parkinson’s disease (PD) pathology through various epidemiological and physiological studies. In animal models, a high-fat diet (HFD) with greater intake of lipid-derived calories leads to accelerated disease onset and progression. The underlying molecular mechanisms of HFD-induced aggravated pathology, however, remain largely unclear. In this study, we aimed to further illuminate the effects of a fat-enriched diet in PD by examining the brainstem and hippocampal transcriptome of alpha-synuclein transgenic mice exposed to a life-long HFD. Investigating individual transcript isoforms, differential gene expression and co-expression clusters, we observed that transcriptional differences between wild-type (WT) and transgenic animals intensified in both regions under HFD. Both brainstem and hippocampus displayed strikingly similar transcriptomic perturbation patterns. Interestingly, expression differences resulted mainly from responses in WT animals to HFD, while these genes remained largely unchanged or were even slightly oppositely regulated by diet in transgenic animals. Genes and co-expressed gene groups exhibiting this dysregulation were linked to metabolic and mitochondrial pathways. Our findings propose the failure of metabolic adaptions as the potential explanation for accelerated disease unfolding under exposure to HFD. From the identified clusters of co-expressed genes, several candidates lend themselves to further functional investigations.

## Introduction

Parkinson’s disease (PD) is a complex neurodegenerative disease with the demise of dopaminergic neurons in the *substantia nigra* leading to dopamine depletion, which, in turn, causes the characteristic clinical phenotype with progressive motility impairment with resting tremor and postural instability (1). While rare familial forms of the disease exist, the preponderance of PD cases occurs seemingly sporadic. Familial as well as sporadic forms share misfolded amyloid fibrils of alpha-synuclein (αSYN) that are accumulated in Lewy bodies, the pathological hallmark of the disease (2).

For αSYN, encoded by the *SNCA* locus, rare point mutations (3–7) and genomic multiplications have been identified in familial cases (8). In addition, several single nucleotide polymorphisms in *SNCA* have been associated with sporadic cases in genome-wide association as well as candidate gene studies (9–15).

Besides genetic contributions and age as key risk factor, an array of environmental factors and lifestyle choices seem to modulate the risk for PD (16). Diet is one of these factors that may—positively and negatively—impact the onset and progression of the disease (17). In humans, epidemiological studies suggest a higher risk for PD among individuals with greater intake of total animal fat (18–21), whereas other studies find no significant associations (19,22–24). These controversial results are also addressed in a recent meta-analysis that suggests high total energy intake rather than total fat intake being relevant for an increased risk of PD (25).

In animal models, the commonly used high-energy diets are so-called HFDs (26) with lipid-derived calories from saturated fat (lard), more unsaturated fat as well as increased levels of sucrose. It is important to note that an HFD is known to induce insulin resistance and to mimic diabetes mellitus type 2 in animal models, which, in humans, is associated with a more aggressive PD phenotype (27–29). Studies in toxin-induced PD models show HFD to exacerbate PD progression by exhibiting increased dopamine depletion in the *substantia nigra*, striatum and nigrostriatal pathway and an aggravation of vascular pathology (30–33). Furthermore, in a genetic mouse model expressing the human mutant h[A30P]αSYN (34), long-term HFD accelerates the onset of the locomotor phenotype, accompanied by earlier alpha-synucleinopathy and astrogliosis (35).

While several hypotheses have been proposed as to how HFD and/or diabetic conditions aggravate PD pathology, including increased oxidative stress, mitochondrial dysfunction and neuroinflammation (reviewed in (36)), the molecular and cellular mechanisms underlying these effects remain to be elucidated. To better understand associated pathways, we here investigated the brainstem and hippocampal transcriptome of αSYN transgenic mice (TG) exposed to a life-long HFD.

## Results

### HFD-dependent differential expression in brainstem and hippocampus

**Figure 1:**
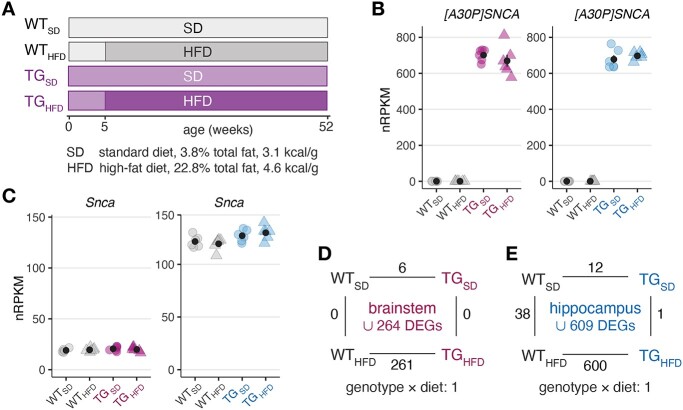
Diet-dependent gene expression changes in brainstem and hippocampus of WT and TG mice. (**A**) Schematic diagram showing the experimental design and groups of WT and TG mice fed either an SD or a HFD from weaning till 12 months of age. From six animals per group, RNA was isolated from hippocampus and brainstem and subjected to RNA-sequencing. (**B**) Expression levels of human *SNCA* in nRPKM shown for brainstem (left) and hippocampus (right) as individual data points for each animal with the mean ± standard error of mean (SEM) per experimental group. (**C**) Expression levels of murine *Snca* for brainstem (left) and hippocampus (right) per animal as individual nRPKM data points with the mean ± SEM. (**D**) Number of DEGs for the main contrasts in brainstem between experimental groups in a 2 × 2 factorial design. Gene expression was modeled as a function of genotype, diet and their interaction. Union of DEGs indicated at the center. (**E**) Analogous to (D) with DEG counts for hippocampus.

Analogous to the experimental paradigm used in our previous study (35), h[A30P]αSYN (TG) as well as wild-type (WT) mice were fed either a standard chow diet (SD, 3.8% total fat, 3.1 kcal/g) or an HFD (22.8% total fat, 4.6 kcal/g) from weaning till 12 months of age (Fig. 1A). As in our previous study, at 12 months of age, mice on HFD weighed about 50 g (48 – 61 g) in contrast to 35 g (33 – 41 g) on a standard diet (SD). For both diets, there was no difference in weight between TG and WT mice. Afterwards, RNA from brainstem and hippocampus was collected from six animals per group and subjected to RNA-sequencing (RNA-seq). After alignment and stringent quality control, the principal component analysis showed no outliers in any of the experimental groups (Supplementary Material, Fig. S1). We further computationally estimated the cell-type composition across samples in order to assess such confounding effects underlying differential expression. Cell type-specific expression profiles based on single-cell reference data (37,38), however, suggested great homogeneity and showed no significant compositional shifts between samples or groups (Supplementary Material, Figs S2 and S3).

After establishing this ground truth in the data, we examined in the first step the expression of endogenous and human *SNCA*. In TG mice, human *SNCA* showed a prominent and similar expression of about 700 normalized Reads Per Kilobase per Million (nRPKM) in both brain regions (Fig. 1C). Endogenous *Snca* expression remained largely unaffected by the transgene, but showed about 10-fold higher levels in hippocampus compared to brainstem (Fig. 1C). Hence, the relative overexpression was much stronger in brainstem, potentially contributing to the pronounced pathology observed in the region (34).

In a next step, expression changes along all primary contrasts were determined resulting in 264 differentially expressed genes (DEGs) for brainstem and 609 for hippocampus (Fig. 1D and E). Besides *SNCA*, few genes showed diet-independent differential expression in TG mice, among them *Auh* and *Gabra2* in brainstem as well as *Cntn3* and *Zfp932* in hippocampus (Supplementary Material, Fig. S4). In contrast, the much larger fraction of DFGs was identified in both brain regions when mice were fed the HFD (Fig. 1D and E), suggesting interactions between genotype and diet. As partially captured directly through the interaction term of the statistical model for genes like *Ppp1r14a* and *Grip2* in brainstem and hippocampus, respectively, these interactions seemingly encompassed lacking or even opposite responses in TG_HFD_ animals compared to WT_HFD_ (Supplementary Material, Fig. S4E and F).

### Differential expression results mainly from genotype–diet interactions

**Figure 2:**
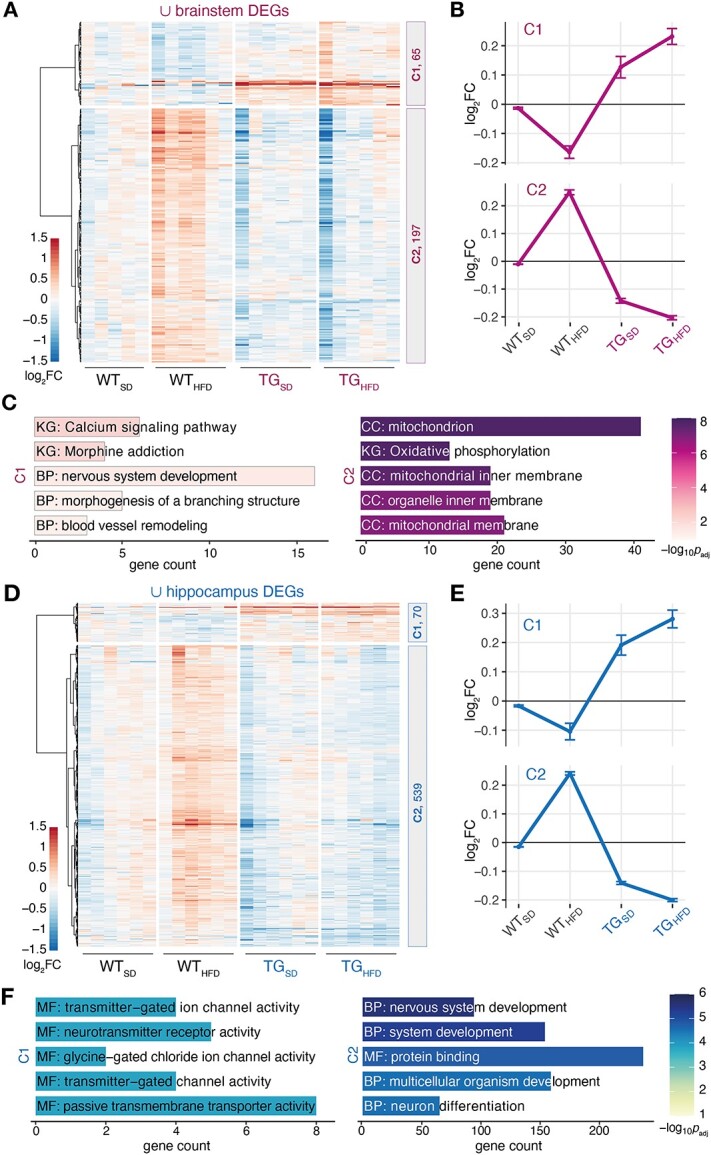
Genotype–diet interactions led to highly similar gene expression patterns in brainstem and hippocampus. (**A**) Heatmap of hierarchically clustered expression profiles (*log_2_* expression change relative to WT_SD_) of 262 DEGs (union of brainstem DEGs indicated in Fig. 1E) across all experimental groups. Number of DEGs in clusters C1 and C2 indicated on the right. (**B**) Average expression profile and standard deviation of C1 and C2 across experimental groups in brainstem. (**C**) Enriched pathways (Gene Ontology, KEGG, Reactome) among C1 (left) and C2 (right) genes. Five most significant terms, their adjusted *P*-values and DEG count are shown. KG: KEGG; BP: biological process, CC: cellular component, MF: molecular function (**D**–**F**) analogues to (A–C) for hippocampus.

In order to put expression changes into perspective, we visually examined the profiles of differential genes from each contrast across the groups (Supplementary Material, Fig. S5). As also apparent from the union of these DEGs, there were two dominating expression patterns that partitioned into clusters C1 and C2 (Fig. 2A and D). Interestingly, this applied to both brain regions in striking similarity. In cluster C1, genes showed lower expression in the WT_HFD_ group but higher in TG animals, largely irrespective of the diet (Fig. 2B and E). Nearly mirror-imaged, genes in cluster C2 showed higher expression in WT_HFD_ mice but lower expression in TG mice, again largely irrespective of diet, a pattern already alluded through genes identified earlier (Supplementary Material, Fig. S4E and F).

In brainstem, C1 and C2 genes were most significantly enriched for *calcium signaling pathway* and *mitochondrion*, respectively (Fig. 2C), whereas C1 and C2 genes in hippocampus were overrepresented for *transmitter-gated ion channel activity* and *nervous system development*, respectively (Fig. 2F).

### Transcriptome perturbations through genotype–diet interactions encompass shifts in transcript isoform composition

In order to explore whether dysregulation in TG animals also extended to other aspects of the transcriptome, we next investigated alternative splicing. Detecting alternatively spliced parts in transcripts still is inherently difficult from RNA-seq data because reads are typically much shorter than the transcripts themselves. While a greater sequencing depth, larger sample size and suitable statistical methods allow mitigating this obstacle, available tools still vary considerably in accuracy and robustness of results (39). Since here, the hippocampal samples were sequenced deeper, we restricted the splicing analyses to this brain region and applied two complementary tools to identify robust effects.

**Figure 3:**
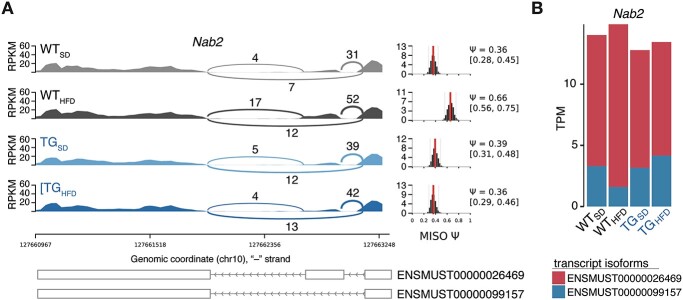
Genotype–diet dependent perturbations extend to transcript isoform usage. (**A**) Sashimi plot illustrating the transcript isoform structure and expression of *Nab2* toward the 3′ end. Depicted are per-group read coverage and read count spanning junctions around exon 6. Coordinates of affected transcript isoforms at the bottom. *MISO*-estimated per cent spliced-in (PSI/Ψ) values with 95% confidence intervals to the right. (**B**) Transcript isoform-specific expression levels of *Nab2* in hippocampal samples. Plotted are the mean TPM per group obtained with *Salmon*.

In line with the gene count for differential expression, the largest number of differential splicing events identified by both tools was observed comparing TG_HFD_ with WT_HFD_ animals (Supplementary Material, Fig. S6A). Among them was *Nab2*, a gene for which HFD led to increased usage of exon 6 (of seven) in WT but not in TG animals (Fig. 3A, Supplementary Material, Fig. S6B). An increased usage of exon 6 agreed with a shift toward higher expression of the longer transcript isoform ENSMUST00000026469 over the shorter ENSMUST00000099157 in WT_HFD_ animals (Fig. 3B). Intriguingly, the shift in transcript composition without entailing a significant gene-level expression change was also observed in brainstem (Supplementary Material, Fig. S6C).

These results indicated that in both brainstem and hippocampus, interactions between genotype and diet gave rise to highly similar dysregulation patterns on a gene as well as transcript level.

### Co-expression analyses point at a shared regulatory network in brainstem and hippocampus

The similarity of perturbances in both brain regions under HFD in WT and TG animals—from individual transcript isoforms to general expression patterns—led us to assume that underlying regulatory principles might be shared between brainstem and hippocampus. Co-expression analyses lend themselves to such investigations as they identify expression similarities between genes beyond comparing DEGs with little information regarding expression relations between them. In co-expression analyses, groups of genes with similar expression patterns across conditions are derived that likely function in the same pathways or regulate the same biological processes, the so-called guilt-by-association principle (40).

**Figure 4:**
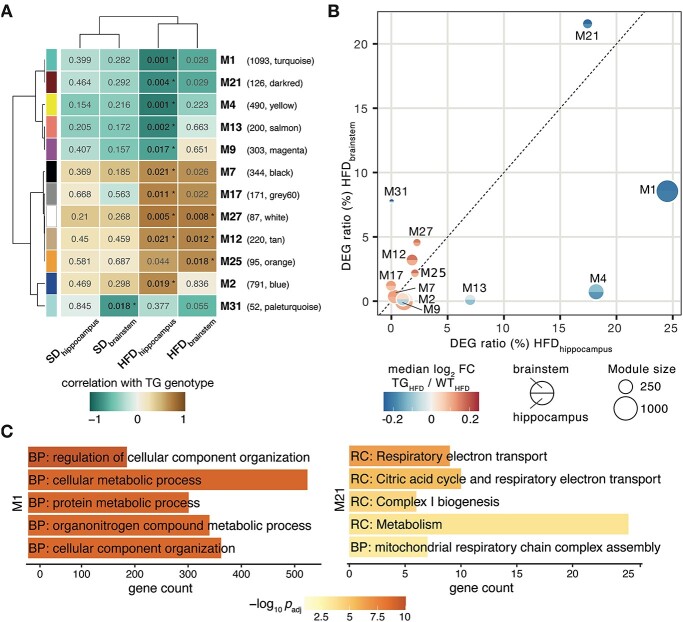
Co-expression analysis identifies gene modules with significant diet-dependent genotype correlations that are highly similar across brain regions. (**A**) Diet-dependent genotype correlations for brainstem and hippocampus with corresponding *P*-values for all significant modules. Cells are color-coded according to the correlation between a module’s expression and the TG genotype. Numbers in cells indicate the nominal *P*-value that is marked with an asterisk when still significant after FDR 0.05 correction. Module colors to the left as per WGCNA partitioning (Supplementary Material, Fig. S7) and their gene count on the right. (**B**) Significant co-expression modules plotted according to their relative DEG content with respect to TG_HFD_/WT_HFD_ in hippocampus (*x*-axis) and brainstem (*y-*axis). Median module expression change color-coded per brain region with the diameter reflecting the total gene count. (**C**) Enriched pathways (Gene Ontology, KEGG, Reactome) for genes in modules M1 (left) and M21 (right). Five most significant terms, their adjusted *P*-values and DEG counts are shown. BP: biological process; RC: Reactome.

Here, we used weighted gene correlation network analysis (WGCNA) (41) to explore co-expression towards identifying gene modules and hub genes underlying the dysregulated expression in TG_HFD_ mice. As we examined different brain regions and dietary conditions, we opted for a consensus analysis that allows deriving co-expression modules across data sets. Following this approach, the consensus network partitioned the expression space into 32 modules (Supplementary Material, Fig. S7). Of those, 12 correlated significantly with the genotype in at least one brain region for either diet (Fig. 4A). Consistent with results from the differential analysis, the correlation was typically stronger under HFD and very similar overall between the brain regions (Fig. 4A). By considering the DEG ratio in the modules, M1 and M21 stood out from the rest as they captured the strongest expression changes (Fig. 4B). Although 1093 genes in module M1 were enriched for metabolic processes, 126 genes in M21 pointed toward mitochondrial pathways (Fig. 4C).

**Figure 5:**
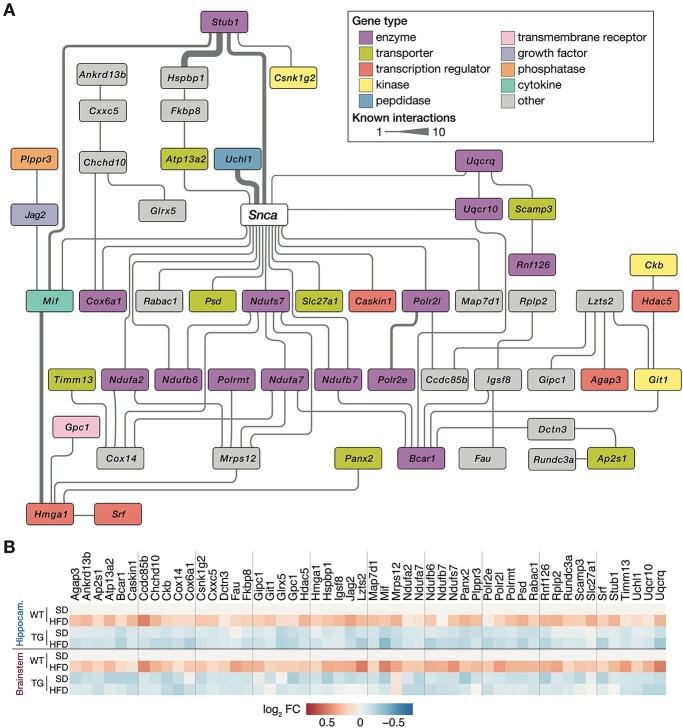
Interaction network of differentially expressed hub genes around *Snca*. (**A**) Network of *Snca* and 57 differentially expressed as well as highly ranked (top 20%) co-expressed genes from modules M1 and M21. Genes color-coded according to their type/function. Line width indicating number of curated interactions between each gene pair. Information derived from IPA. (**B**) Expression changes (as *log_2_* fold changes per group relative to WT_SD_) for 57 alphabetically sorted network genes in (A) for hippocampus and brainstem.

Following the reasoning of WGCNA that higher ranked genes and their relations within the modules are biologically more meaningful (42), we focused on the top 20% of genes in M1 and M21 that were differentially expressed. From the resulting 150 genes, more than one-third were linked to the *SNCA* interactome (Fig. 5A), indicating that the filtering steps led to relevant αSYN-related biology. Intriguingly, for all these genes, WT mice responded to HFD, whereas TG animals showed no or even opposite regulation (Fig. 5B). This failure of adaptation to HFD apparent in different brain regions might explain the previously described accelerated pathology in TG mice under long-term HFD (35).

## Discussion

As shown in our previous study, mice expressing human mutant αSYN (TG) as a model for PD have increased neuroinflammation as well as a significantly accelerated onset of neurodegeneration and terminal phenotype under a lifelong HFD (35). Towards a better molecular understanding of the interactions between genetic predisposition and diet, we here profiled the transcriptome in brainstem and hippocampus of this mouse model. Intriguingly, we observed a transcriptional adaptation of WT animals in response to an HFD that was largely missing in TG animals. Thereby, both brain regions exhibited great similarity in transcriptomic perturbations, ranging from individual transcript isoforms, to common differential genes and overall patterns of expression. Differential as well as co-expression analyses highlighted dysregulated genes that are involved in metabolic processes and mitochondria-related pathways.

The enrichment of perturbed genes for metabolic pathways agrees with epidemiological and physiological studies in PD research (43–45). Besides dietary effects that possibly modulate disease risk and influence development, disturbances in metabolic processes are also observed in the course of the disease (46).

With respect to mitochondria, our results agree with numerous findings in animal models as well as patients that suggest a pivotal role of mitochondrial dysfunction in PD pathophysiology (47). The central role of mitochondria-related processes was recognized early on as PD patients show a decreased activity of the mitochondria electron transport chain, primarily complex I. This dysfunction is thought to lead not only to increased generation of mitochondrial-derived reactive oxygen species and subsequent oxidative damage but also to the energy failure because of the inability of neurons to compensate the lack of ATP generation. Such bioenergetic perturbations are thought to be central in neurodegeneration as they are linked directly to important homeostatic processes in dopaminergic cells such as neurotransmitter release, axonal vesical transport, protein quality control and cell metabolism (46).

Our study points at a decrease of mitochondrial gene expression in the context of PD that is well reflected on protein level. Focus has, for example, been put on defective mitochondrial protein import showing reduced protein levels of the mitochondrial translocases TIM23 and TOM20 as well as nuclear-encoded mitochondrial proteins such as NDUFS3 and COX IV in *post-mortem substantia nigra* samples of PD patients (48). In this context. αSYN seems to play a crucial role as the aberrant αSYN–TOM20 interaction in nigrostriatal dopaminergic neurons is associated with such loss of imported mitochondrial proteins (49), thereby confirming decreased protein levels in the human disease.

Besides the relation between mitochondrial (dys-)function and PD, there are also links between mitochondria and HFD. Typically, HFDs are known for a negative impact on mitochondrial biogenesis, dynamics and function that, ultimately, lead to glucose intolerance and insulin resistance (50). However, feeding on HFD has also been shown to increase protein levels of mitochondrial genes in muscle tissue despite inducing glucose intolerance and insulin resistance (51). A number of earlier studies provide further evidence that HFD has beneficial effects on mitochondria (52–55) and promotes their biogenesis with increased protein levels of several mitochondrial genes as well as their oxidative capacity of fatty acids in skeletal muscle (56). Hence, some of the observed expression changes in WT_HFD_ might reflect adaptations of the metabolic regulatory network in the cell to cope with altered fatty acid levels.

In particular, we observed increased expression of several mitochondrial genes in WT_HFD_ animals such as *Ndufa2*, *Cox6a1*, *Uqcrq*. These genes were slightly decreased in TG_SD_ mice already and, in contrast to WT animals, showed no increase in TG animals under HFD.

Besides genes directly involved in mitochondrial function, others previously linked to *SNCA* also showed failed adaptation in TG_HFD_ mice including *Stub1*, *Uchl1*, *Atp13a2*/*Park9* and *Mif*. Interestingly, increase of these proteins is typically considered protective in the context of PD. Hence, lower expression of these genes as observed in TG_HFD_ animals might contribute to the detrimental phenotype of these mice (35).

*Stub1* encodes an E3 ubiquitin-protein ligase, also known as CHIP in human, which targets misfolded chaperone substrates toward proteasomal degradation. A beneficial role of CHIP in the pathogenesis of several neurological diseases has been shown (57). CHIP co-localizes with αSYN in Lewy bodies and inclusions, and its overexpression reduces αSYN aggregation and increases its degradation via proteasomal as well as lysosomal degradation pathways (58). Moreover, CHIP preferentially binds and degrades toxic oligomeric forms of αSYN (59) and ubiquitinates αSYN itself (60).

*Uchl1*, also known as *PARK5* in human, encodes a ubiquitin-protein hydrolase that plays an important role in maintaining a stable pool of monoubiquitin for ubiquitination reactions. Mutations of the gene are associated with PD and its protein is found in Lewy bodies (61,62). It has been proposed that modulation of UCHL1 activity may serve as a therapeutic tool to enhance the autophagy pathway and induce clearance of aggregated αSYN (63). Here, failure of TG mice to increase *Uchl1* expression under HFD agrees with the worse phenotype (35).

A similar hypothesis can be proposed for *Atp13a2* or *PARK9* in human, which regulates the autophagy–lysosome pathway and plays a role in lipid homeostasis. ATP13A2 promotes the secretion of exosomes as well as secretion of αSYN via exosomes (64,65) and its overexpression suppresses toxicity of αSYN.

Lastly, *Mif*, encoding the macrophage migration inhibitory factor, is a pluripotent pro-inflammatory cytokine. MIF reduces apoptosis and induces autophagy in an *in vitro* model of PD and, thereby, might mediate neuroprotective effects in PD (66).

For these example genes, the expression increase in WT_HFD_ animals potentially helps to cope with challenges imposed on the cell through higher energy levels. It is known that a HFD results in neurochemical adaptations including altered neurotransmission and bioenergetics in the hippocampus of rodents (67). The failure to adaptation in TG_HFD_ mice might be a key principle that explains the aggravated phenotype.

Some of the highlighted genes such as *Stub1* and *Uchl1* are linked to ubiquitination and autophagy mechanisms and point at a crucial relationship between these mechanisms as well as metabolic- or mitochondria-related pathways. The quality of mitochondria regulating numerous metabolic pathways is known to be strictly monitored to maintain cell homeostasis (68). Impaired mitochondrial quality is readily identified to eliminate damaged mitochondria, a process relying on the orchestrated crosstalk between ubiquitin signaling and autophagy. The loss of mitochondrial quality control systems is known to be associated with many types of neurodegenerative diseases including PD (68). In PD, progressive accumulation of dysfunctional mitochondria ultimately impairs cellular metabolism and causes neuronal death. As there is increasing evidence that energy metabolism plays a fundamental role in the pathomechanism of neurodegenerative diseases (69–73), it is recently becoming a potential target for preventing and treating PD (72). However, the notion of PD as a disease initiated by dysfunctions of energy metabolism just barely started (72). Our approach highlights the failure of adaptations to metabolic challenges in the context of PD and points at candidate genes that might be addressed therapeutically.

However, although these candidate genes seem plausible and the transcriptomic analyses pointed at affected pathways in the αSYN interactome, they do not allow identifying an entry point for the perturbations or deriving further causalities between genes. Such functional aspects need to be addressed by future studies. We are, however, convinced our results provide a basis to select targets for such investigations.

Taken together, our work shows that a long-term HFD leads to gene expression adaptations of several genes linked to mitochondrial and metabolic biology in the brain of WT mice. In αSYN TG animals, in contrast, there is a widespread failure of these diet-induced transcriptional adaptations, indicating that the previously observed aggravation of phenotype might be based on a failed response of related genes.

## Materials and Methods

### Animals and diets

TG of C57BL/6 background expressing human mutant h[A30P]αSYN under the control of the CNS neuron-specific Thy1 promotor (34,74) were maintained as a homozygous colony. WT controls were derived from the same transgenic outcross with C57BL/6 mice and maintained as a parallel colony. Only male mice were used in the study. For grouping the mice into either a standard or HFD, we used randomization within blocks representing litters. Thereby, we sought to mitigate any litter bias due to genetic effects that might confound differential expression analyses later on. From the age of 5 weeks onward till 12 months of age, homozygous (Thy1)-h[A30P]AS and WT mice were either kept on standard chow diet (SD, *n* = 56) (3.8% total fat, 3.1 kcal/g, ssniff R/M H Extrudat; ssniff Spezialitäten GmbH, Soest, Germany) or HFD (22.8% total fat, 4.6 kcal/g, TD.06415 Adjusted Calories Diet 45/Fat; Teklad Custom Research Diets, Harlan Laboratories, Boxmeer, The Netherlands). Groups of three to four male mice were housed in standard cages (365 × 207 × 140 mm, Typ II long) with normal light/dark cycle (12 h light/12 h dark) and free access to food and water. To avoid gene expression changes during the preparation process, WT and TG mice were sacrificed with cervical dislocation followed by head decapitation within 2 min from disturbing the home cage. Brain regions were immediately dissected on ice and snap frozen in liquid nitrogen. All animal procedures were approved by local government authorities for animal research (file references N13/16) according to the guidelines for laboratory animal care.

### RNA isolation and sequencing

To focus on the same regions as before when describing the pathology in the animal model (35), the entire brainstem more precisely the *medulla* (*oblongata* and *spinalis*) *posterior* to approximately bregma −8 based on a coronal view was prepared (https://mouse.brain-map.org/experiment/thumbnails/100048576?image_type=atlas). For the hippocampus, the entire structure including all subfields was dissected. Total RNA and DNA from brainstem and hippocampus (*n* = 6 animals for each of the four experimental groups per brain region) were simultaneously extracted using the *AllPrep DNA/RNA Mini Kit* (Qiagen) using the manufacturer’s protocol. Quality was assessed with an *Agilent 2100 Bioanalyzer*. Samples with high RNA integrity number (>8) were selected for library construction; one WT_SD_ sample in brainstem failed this criterion and was not included. Using the *TruSeq RNA Sample Prep Kit* (Illumina), poly(A)-selected single-end sequencing libraries (75 bp read length) were generated according to the manufacturer’s instructions. To normalize the volume and amount of each brain regions per animal, 500 ng of the total RNA per sample was used. All libraries were sequenced on an Illumina HiSeq 2500 platform at a depth of 10–15 million reads each. Library preparation and sequencing were performed by the same individual using a design to minimize the batch effects.

To meet blinding strategies throughout the experimental procedure, the experimenters for brain dissection, RNA isolation, library preparation and sequencing were unaware of the animal’s group during experimentation.

### Quality control, alignment and expression analysis

Read quality of RNA-seq data was assessed using *FastQC* (v0.11.9) (75) to identify sequencing cycles with low average quality, adaptor contamination or repetitive sequences from PCR amplification. Reads were aligned using *STAR* (v2.7.9a) (76) allowing gapped alignments to account for splicing against a custom-built genome composed of the Ensembl *Mus musculus* genome v104 and the human *SNCA* transgene. Normalized read counts for all genes were obtained using *DESeq2* (v1.32.0) (77). Transcripts covered with <50 reads were excluded from the analysis leaving 13 309 genes in brainstem and 13 251 in hippocampus for determining differential expression in each of the primary contrasts between experimental groups.

The 2 × 2 factorial design of the experiment was captured in a generalized linear model in *DESeq2* modeling expression as a function of genotype, diet and their interaction. Surrogate variable analysis (*sva*, v3.40.0) was used to minimize unwanted variation between samples (78). Given that differences in transcript abundances in brain tissue are often small in magnitude and *in vivo* RNA-seq data are deemed to be more variable (79), we set |*log_2_* fold-change | ≥ 0.3 and adjusted *P*-value ≤ 0.05 to determine DFGs.

Gene-level abundances were derived from *DESeq2* as normalized read counts and used for calculating the *log_2_*-transformed expression changes underlying expression heatmaps and *k*-means clustering with ratios computed relative to the mean expression in WT_SD_. *Thy1* and human *SNCA* were excluded here because of their unique and strongly biased profiles.

The *sizeFactor*-normalized counts provided by *DESeq2* also calculated nRPKMs total reads as a measure of relative gene expression as motivated before (80). Transcript-level expression was determined using *Salmon* (v1.5.2, parameters: numGibbsSamples 20, seqBias, gcBias, validateMappings) (81). Transcripts per million (TPM) values obtained with *Salmon* were scaled (*scaleInfReps*) using the *tximeta* (v1.10.0) R package (82). In addition, *JunctionSeq* (v1.21.0, default parameters) (39) was used to identify alternative splicing events underlying transcript-level changes.

To identify alternative splicing events (alternative 5′ and 3′ splice site, retained intron, skipped exon, mutually exclusive exon), *MISO* (v0.5.4) was used for merged hippocampal samples and filtered for events with --num-total 200 --num-inc 10 --num-exc 10 --num-sum-inc-exc 20 --delta-psi 0.2 --bayes-factor 10 (83,84).

*Gprofiler2* (v.0.2.1) (85) with *P*_FDR_ ≤ 0.05 was used to determine functional enrichments among gene sets against Gene Ontology, KEGG and Reactome. *Thy1* and human *SNCA* were excluded when determining enrichments.

Interactions among genes were derived from curated data in *Ingenuity Pathway Analysis* (IPA, v01–20-04, Qiagen) and visualized in *Cytoscape* (86).

### Co-expression analysis

Four sets were formed, each containing the expression data from hippocampal or brainstem samples exposed to SD or HFD, respectively. WGCNA (41) was used to establish a consensus network in order to identify gene co-expression across all four data sets. WGCNA was based on pairwise correlation between all gene pairs in each data set. As the correlation method, biweight midcorrelation (87) was used with *maxPOutliers* = 0.1, thereby minimizing the influence of potential outliers. Correlations were transformed in a signed hybrid similarity matrix where negative and zero correlations equal to zero, whereas positive correlations remain unchanged. To generate the network adjacency, the similarity matrix was raised to the power *β* = 8, the minimum value that approximated a scale-free topology in all data sets, thus suppressing low correlations. For a measure of interconnectedness, adjacency was transformed into a topological overlap measure (TOM) that is informed by the adjacency of every gene pair plus the connection strength they share with the neighboring genes. Before calculating the consensus TOM (cTOM), the individual TOMs were calibrated using a 0.95-single quantile scaling. TOMs were raised to a power such that the 95th percentile of all other data sets equaled the same quantile of the reference set (hippocampus under SD). Thus, potential bias deriving from different statistical properties were mitigated best. cTOM was created by selecting the component-wise 0th quantile of the individual TOMs, meaning that for each gene pair, the minimum TOM value across all sets was given as input. A hierarchical clustering of a TOM-based dissimilarity measure (1-cTOM) was used to define modules by applying the *Dynamic Tree Cut* algorithm (88). Each of these modules was summarized by its eigengene, providing a single value for a module’s expression profile. Based on the eigengene correlation matrix, final modules were derived by iteratively clustering eigengenes based on dissimilarity (given by one minus the respective correlation) and cutting the resulting dendrogram at height 0.1, causing all modules with eigengene correlation ≥ 0.9 to be merged. Finally, module eigengenes were correlated with a dichotomous genotype trait to identify modules affected by transgenic effects. A joint Bayesian-frequentistic algorithm combining the Bayes factor (BF) (89) and significance of a correlation was used to identify modules associated with the disease status. Modules with an eigengene-trait correlation with *P*_FDR_ ≤ 0.05 | BF ≥ 3 were considered significantly associated with genotype status.

## Supplementary Material

SuppFigures_ddac205

## Data Availability

RNA-seq data files have been uploaded to GEO database and are available under the accession number GSE197511.

## References

[1] Dauer, W. and Przedborski, S. (2003) Parkinson’s disease: mechanisms and models. Neuron, 39, 889–909. 12971891 10.1016/s0896-6273(03)00568-3

[2] Spillantini, M.G., Schmidt, M.L., Lee, V.M.-Y., Trojanowski, J.Q., Jakes, R. and Goedert, M. (1997) α-Synuclein in Lewy bodies. Nature, 388, 839–840. 9278044 10.1038/42166

[3] Krüger, R., Kuhn, W., Müller, T., Woitalla, D., Graeber, M., Kösel, S., Przuntek, H., Epplen, J.T., Schöls, L. and Riess, O. (1998) Ala30Pro mutation in the gene encoding α-synuclein in Parkinson’s disease. Nat. Genet., 18, 106–108. 9462735 10.1038/ng0298-106

[4] Lesage, S., Anheim, M., Letournel, F., Bousset, L., Honoré, A., Rozas, N., Pieri, L., Madiona, K., Dürr, A., Melki, R. et al. (2013) G51D α-synuclein mutation causes a novel parkinsonian-pyramidal syndrome. Ann. Neurol., 73, 459–471. 23526723 10.1002/ana.23894

[5] Polymeropoulos, M.H., Lavedan, C., Leroy, E., Ide, S.E., Dehejia, A., Dutra, A., Pike, B., Root, H., Rubenstein, J., Boyer, R. et al. (1997) Mutation in the α-synuclein gene identified in families with Parkinson’s disease. Science, 276, 2045–2047. 9197268 10.1126/science.276.5321.2045

[6] Proukakis, C., Dudzik, C.G., Brier, T., MacKay, D.S., Cooper, J.M., Millhauser, G.L., Houlden, H. and Schapira, A.H. (2013) A novel α-synuclein missense mutation in Parkinson disease. Neurology, 80, 1062–1064. 23427326 10.1212/WNL.0b013e31828727ba PMC3653201

[7] Zarranz, J.J., Alegre, J., Gómez-Esteban, J.C., Lezcano, E., Ros, R., Ampuero, I., Vidal, L., Hoenicka, J., Rodriguez, O., Atarés, B. et al. (2004) The new mutation, E46K, of α-synuclein causes Parkinson and Lewy body dementia. Ann. Neurol., 55, 164–173. 14755719 10.1002/ana.10795

[8] Chartier-Harlin, M.-C., Kachergus, J., Roumier, C., Mouroux, V., Douay, X., Lincoln, S., Levecque, C., Larvor, L., Andrieux, J., Hulihan, M. et al. (2004) α-Synuclein locus duplication as a cause of familial Parkinson’s disease. Lancet, 364, 1167–1169. 15451224 10.1016/S0140-6736(04)17103-1

[9] Consortium, U.K.P.S.D., Wellcome Trust Case Control, C, Spencer, C.C., Plagnol, V., Strange, A., Gardner, M., Paisan-Ruiz, C., Band, G., Barker, R.A., Bellenguez, C. et al. (2011) Dissection of the genetics of Parkinson’s disease identifies an additional association 5′ of SNCA and multiple associated haplotypes at 17q21. Hum. Mol. Genet., 20, 345–353. 21044948 10.1093/hmg/ddq469 PMC3005904

[10] Edwards, T.L., Scott, W.K., Almonte, C., Burt, A., Powell, E.H., Beecham, G.W., Wang, L., Zuchner, S., Konidari, I., Wang, G. et al. (2010) Genome-wide association study confirms SNPs in SNCA and the MAPT region as common risk factors for Parkinson disease. Ann. Hum. Genet., 74, 97–109. 20070850 10.1111/j.1469-1809.2009.00560.x PMC2853717

[11] Miyake, Y., Tanaka, K., Fukushima, W., Kiyohara, C., Sasaki, S., Tsuboi, Y., Yamada, T., Oeda, T., Shimada, H., Kawamura, N. et al. (2012) SNCA polymorphisms, smoking, and sporadic Parkinson’s disease in Japanese. Parkinsonism Relat. Disord., 18, 557–561. 22425546 10.1016/j.parkreldis.2012.02.016

[12] Mueller, J.C., Fuchs, J., Hofer, A., Zimprich, A., Lichtner, P., Illig, T., Berg, D., Wullner, U., Meitinger, T. and Gasser, T. (2005) Multiple regions of alpha-synuclein are associated with Parkinson’s disease. Ann. Neurol., 57, 535–541. 15786467 10.1002/ana.20438

[13] Nalls, M.A., Pankratz, N., Lill, C.M., Do, C.B., Hernandez, D.G., Saad, M., DeStefano, A.L., Kara, E., Bras, J., Sharma, M. et al. (2014) Large-scale meta-analysis of genome-wide association data identifies six new risk loci for Parkinson’s disease. Nat. Genet., 46, 989–993. 25064009 10.1038/ng.3043 PMC4146673

[14] Saad, M., Lesage, S., Saint-Pierre, A., Corvol, J.C., Zelenika, D., Lambert, J.C., Vidailhet, M., Mellick, G.D., Lohmann, E., Durif, F. et al. (2011) Genome-wide association study confirms BST1 and suggests a locus on 12q24 as the risk loci for Parkinson’s disease in the European population. Hum. Mol. Genet., 20, 615–627. 21084426 10.1093/hmg/ddq497

[15] Simon-Sanchez, J., Schulte, C., Bras, J.M., Sharma, M., Gibbs, J.R., Berg, D., Paisan-Ruiz, C., Lichtner, P., Scholz, S.W., Hernandez, D.G. et al. (2009) Genome-wide association study reveals genetic risk underlying Parkinson’s disease. Nat. Genet., 41, 1308–1312. 19915575 10.1038/ng.487 PMC2787725

[16] Marras, C., Canning, C.G. and Goldman, S.M. (2019) Environment, lifestyle, and Parkinson’s disease: implications for prevention in the next decade. Mov. Disord., 34, 801–811. 31091353 10.1002/mds.27720

[17] Seidl, S.E., Santiago, J.A., Bilyk, H. and Potashkin, J.A. (2014) The emerging role of nutrition in Parkinson’s disease. Front. Aging Neurosci., 6, 36. 24639650 10.3389/fnagi.2014.00036 PMC3945400

[18] Anderson, C., Checkoway, H., Franklin, G.M., Beresford, S., Smith-Weller, T. and Swanson, P.D. (1999) Dietary factors in Parkinson’s disease: the role of food groups and specific foods. Mov. Disord., 14, 21–27. 9918340 10.1002/1531-8257(199901)14:1<21::aid-mds1006>3.0.co;2-y

[19] Chen, H., Zhang, S.M., Hernan, M.A., Willett, W.C. and Ascherio, A. (2003) Dietary intakes of fat and risk of Parkinson’s disease. Am. J. Epidemiol., 157, 1007–1014. 12777364 10.1093/aje/kwg073

[20] Johnson, C.C., Gorell, J.M., Rybicki, B.A., Sanders, K. and Peterson, E.L. (1999) Adult nutrient intake as a risk factor for Parkinson’s disease. Int. J. Epidemiol., 28, 1102–1109. 10661654 10.1093/ije/28.6.1102

[21] Logroscino, G., Marder, K., Cote, L., Tang, M.X., Shea, S. and Mayeux, R. (1996) Dietary lipids and antioxidants in Parkinson’s disease: a population-based, case-control study. Ann. Neurol., 39, 89–94. 8572672 10.1002/ana.410390113

[22] Chen, H., Zhang, S.M., Hernan, M.A., Willett, W.C. and Ascherio, A. (2002) Diet and Parkinson’s disease: a potential role of dairy products in men. Ann. Neurol., 52, 793–801. 12447934 10.1002/ana.10381

[23] Hellenbrand, W., Boeing, H., Robra, B.P., Seidler, A., Vieregge, P., Nischan, P., Joerg, J., Oertel, W.H., Schneider, E. and Ulm, G. (1996) Diet and Parkinson’s disease. II: a possible role for the past intake of specific nutrients. Results from a self-administered food-frequency questionnaire in a case-control study. Neurology, 47, 644–650. 8797457 10.1212/wnl.47.3.644

[24] Powers, K.M., Smith-Weller, T., Franklin, G.M., Longstreth, W.T., Jr., Swanson, P.D. and Checkoway, H. (2003) Parkinson’s disease risks associated with dietary iron, manganese, and other nutrient intakes. Neurology, 60, 1761–1766. 12796527 10.1212/01.wnl.0000068021.13945.7f

[25] Qu, Y., Chen, X., Xu, M.M. and Sun, Q. (2019) Relationship between high dietary fat intake and Parkinson’s disease risk: a meta-analysis. Neural Regen. Res., 14, 2156–2163. 31397355 10.4103/1673-5374.262599 PMC6788237

[26] Van Heek, M., Compton, D.S., France, C.F., Tedesco, R.P., Fawzi, A.B., Graziano, M.P., Sybertz, E.J., Strader, C.D. and Davis, H.R., Jr. (1997) Diet-induced obese mice develop peripheral, but not central, resistance to leptin. J. Clin. Invest., 99, 385–390. 9022070 10.1172/JCI119171 PMC507810

[27] Chohan, H., Senkevich, K., Patel, R.K., Bestwick, J.P., Jacobs, B.M., Bandres Ciga, S., Gan-Or, Z. and Noyce, A.J. (2021) Type 2 diabetes as a determinant of Parkinson's disease risk and progression. Mov. Disord., 36, 1420–1429. 33682937 10.1002/mds.28551 PMC9017318

[28] Mollenhauer, B., Zimmermann, J., Sixel-Doring, F., Focke, N.K., Wicke, T., Ebentheuer, J., Schaumburg, M., Lang, E., Friede, T., Trenkwalder, C. et al. (2019) Baseline predictors for progression 4 years after Parkinson’s disease diagnosis in the De Novo Parkinson cohort (DeNoPa). Mov. Disord., 34, 67–77. 30468694 10.1002/mds.27492

[29] Pagano, G., Polychronis, S., Wilson, H., Giordano, B., Ferrara, N., Niccolini, F. and Politis, M. (2018) Diabetes mellitus and Parkinson disease. Neurology, 90, e1654–e1662. 29626177 10.1212/WNL.0000000000005475

[30] Bousquet, M., St-Amour, I., Vandal, M., Julien, P., Cicchetti, F. and Calon, F. (2012) High-fat diet exacerbates MPTP-induced dopaminergic degeneration in mice. Neurobiol. Dis., 45, 529–538. 21971528 10.1016/j.nbd.2011.09.009

[31] Choi, J.Y., Jang, E.H., Park, C.S. and Kang, J.H. (2005) Enhanced susceptibility to 1-methyl-4-phenyl-1,2,3,6-tetrahydropyridine neurotoxicity in high-fat diet-induced obesity. Free Radic. Biol. Med., 38, 806–816. 15721991 10.1016/j.freeradbiomed.2004.12.008

[32] Morris, J.K., Bomhoff, G.L., Stanford, J.A. and Geiger, P.C. (2010) Neurodegeneration in an animal model of Parkinson’s disease is exacerbated by a high-fat diet. Am J Physiol Regul Integr Comp Physiol, 299, R1082–R1090. 20702796 10.1152/ajpregu.00449.2010 PMC2957375

[33] Elabi, O.F., Cunha, J., Gaceb, A., Fex, M. and Paul, G. (2021) High-fat diet-induced diabetes leads to vascular alterations, pericyte reduction, and perivascular depletion of microglia in a 6-OHDA toxin model of Parkinson disease. J. Neuroinflammation, 18, 175. 34376193 10.1186/s12974-021-02218-8 PMC8353816

[34] Kahle, P.J., Neumann, M., Ozmen, L. and Haass, C. (2000) Physiology and pathophysiology of alpha-synuclein. Cell culture and transgenic animal models based on a Parkinson’s disease -associated protein. Ann. N Y Acad. Sci., 920, 33–41. 11193173 10.1111/j.1749-6632.2000.tb06902.x

[35] Rotermund, C., Truckenmuller, F.M., Schell, H. and Kahle, P.J. (2014) Diet-induced obesity accelerates the onset of terminal phenotypes in alpha-synuclein transgenic mice. J. Neurochem., 131, 848–858. 24995537 10.1111/jnc.12813

[36] Sergi, D., Renaud, J., Simola, N. and Martinoli, M.G. (2019) Diabetes, a contemporary risk for Parkinson's disease: epidemiological and cellular evidences. Front. Aging Neurosci., 11, 302. 31787891 10.3389/fnagi.2019.00302 PMC6856011

[37] Liu, W., Venugopal, S., Majid, S., Ahn, I.S., Diamante, G., Hong, J., Yang, X. and Chandler, S.H. (2020) Single-cell RNA-seq analysis of the brainstem of mutant SOD1 mice reveals perturbed cell types and pathways of amyotrophic lateral sclerosis. Neurobiol. Dis., 141, 104877. 32360664 10.1016/j.nbd.2020.104877 PMC7519882

[38] Zeisel, A., Munoz-Manchado, A.B., Codeluppi, S., Lonnerberg, P., La Manno, G., Jureus, A., Marques, S., Munguba, H., He, L., Betsholtz, C. et al. (2015) Brain structure. Cell types in the mouse cortex and hippocampus revealed by single-cell RNA-seq. Science, 347, 1138–1142. 25700174 10.1126/science.aaa1934

[39] Hartley, S.W. and Mullikin, J.C. (2016) Detection and visualization of differential splicing in RNA-Seq data with JunctionSeq. Nucleic Acids Res., 44, e127. 27257077 10.1093/nar/gkw501 PMC5009739

[40] van Dam, S., Vosa, U., van der Graaf, A., Franke, L. and de Magalhaes, J.P. (2018) Gene co-expression analysis for functional classification and gene-disease predictions. Brief. Bioinform., 19, 575–592. 28077403 10.1093/bib/bbw139 PMC6054162

[41] Zhang, B. and Horvath, S. (2005) A general framework for weighted gene co-expression network analysis. Stat. Appl. Genet. Mol. Biol., 4, Article 17. 10.2202/1544-6115.1128 16646834

[42] Langfelder, P. and Horvath, S. (2008) WGCNA: an R package for weighted correlation network analysis. BMC Bioinform., 9, 559. 10.1186/1471-2105-9-559 PMC2631488 19114008

[43] Picca, A., Calvani, R., Landi, G., Marini, F., Biancolillo, A., Gervasoni, J., Persichilli, S., Primiano, A., Urbani, A., Bossola, M. et al. (2019) Circulating amino acid signature in older people with Parkinson’s disease : a metabolic complement to the EXosomes in PArkiNson Disease (EXPAND) study. Exp. Gerontol., 128, 110766. 31666195 10.1016/j.exger.2019.110766

[44] Shao, Y., Li, T., Liu, Z., Wang, X., Xu, X., Li, S., Xu, G. and Le, W. (2021) Comprehensive metabolic profiling of Parkinson’s disease by liquid chromatography-mass spectrometry. Mol. Neurodegener., 16, 4. 33485385 10.1186/s13024-021-00425-8 PMC7825156

[45] van Kessel, S.P. and El Aidy, S. (2019) Bacterial metabolites mirror altered gut microbiota composition in patients with Parkinson's disease. J. Parkinsons Dis., 9, S359–S370. 31609701 10.3233/JPD-191780 PMC6839483

[46] Anandhan, A., Jacome, M.S., Lei, S., Hernandez-Franco, P., Pappa, A., Panayiotidis, M.I., Powers, R. and Franco, R. (2017) Metabolic dysfunction in Parkinson's disease: bioenergetics, redox homeostasis and central carbon metabolism. Brain Res. Bull., 133, 12–30. 28341600 10.1016/j.brainresbull.2017.03.009 PMC5555796

[47] Exner, N., Lutz, A.K., Haass, C. and Winklhofer, K.F. (2012) Mitochondrial dysfunction in Parkinson’s disease: molecular mechanisms and pathophysiological consequences. EMBO J., 31, 3038–3062. 22735187 10.1038/emboj.2012.170 PMC3400019

[48] Franco-Iborra, S., Cuadros, T., Parent, A., Romero-Gimenez, J., Vila, M. and Perier, C. (2018) Defective mitochondrial protein import contributes to complex I-induced mitochondrial dysfunction and neurodegeneration in Parkinson’s disease. Cell Death Dis., 9, 1122. 30405116 10.1038/s41419-018-1154-0 PMC6221944

[49] Di Maio, R., Barrett, P.J., Hoffman, E.K., Barrett, C.W., Zharikov, A., Borah, A., Hu, X., McCoy, J., Chu, C.T., Burton, E.A. et al. (2016) Alpha-synuclein binds to TOM20 and inhibits mitochondrial protein import in Parkinson’s disease. Sci. Transl. Med., 8, 342ra378. 10.1126/scitranslmed.aaf3634 PMC5016095 27280685

[50] Sergi, D., Naumovski, N., Heilbronn, L.K., Abeywardena, M., O'Callaghan, N., Lionetti, L. and Luscombe-Marsh, N. (2019) Mitochondrial (dys)function and insulin resistance: from pathophysiological molecular mechanisms to the impact of diet. Front. Physiol., 10, 532. 31130874 10.3389/fphys.2019.00532 PMC6510277

[51] Hancock, C.R., Han, D.H., Chen, M., Terada, S., Yasuda, T., Wright, D.C. and Holloszy, J.O. (2008) High-fat diets cause insulin resistance despite an increase in muscle mitochondria. Proc. Natl. Acad. Sci. USA, 105, 7815–7820. 18509063 10.1073/pnas.0802057105 PMC2409421

[52] McAinch, A.J., Lee, J.S., Bruce, C.R., Tunstall, R.J., Hawley, J.A. and Cameron-Smith, D. (2003) Dietary regulation of fat oxidative gene expression in different skeletal muscle fiber types. Obes. Res., 11, 1471–1479. 14694211 10.1038/oby.2003.197

[53] Miller, W.C., Bryce, G.R. and Conlee, R.K. (1984) Adaptations to a high-fat diet that increase exercise endurance in male rats. J. Appl. Physiol. Respir. Environ. Exerc. Physiol., 56, 78–83. 6693336 10.1152/jappl.1984.56.1.78

[54] Nemeth, P.M., Rosser, B.W., Choksi, R.M., Norris, B.J. and Baker, K.M. (1992) Metabolic response to a high-fat diet in neonatal and adult rat muscle. Am. J. Phys., 262, C282–C286. 10.1152/ajpcell.1992.262.2.C282 1539619

[55] Simi, B., Sempore, B., Mayet, M.H. and Favier, R.J. (1985) (1991) additive effects of training and high-fat diet on energy metabolism during exercise. J. Appl. Physiol., 71, 197–203. 10.1152/jappl.1991.71.1.197 1917743

[56] Turner, N., Bruce, C.R., Beale, S.M., Hoehn, K.L., So, T., Rolph, M.S. and Cooney, G.J. (2007) Excess lipid availability increases mitochondrial fatty acid oxidative capacity in muscle: evidence against a role for reduced fatty acid oxidation in lipid-induced insulin resistance in rodents. Diabetes, 56, 2085–2092. 17519422 10.2337/db07-0093

[57] Zhang, S., Hu, Z.W., Mao, C.Y., Shi, C.H. and Xu, Y.M. (2020) CHIP as a therapeutic target for neurological diseases. Cell Death Dis., 11, 727. 32908122 10.1038/s41419-020-02953-5 PMC7481199

[58] Shin, Y., Klucken, J., Patterson, C., Hyman, B.T. and McLean, P.J. (2005) The co-chaperone carboxyl terminus of Hsp70-interacting protein (CHIP) mediates alpha-synuclein degradation decisions between proteasomal and lysosomal pathways. J. Biol. Chem., 280, 23727–23734. 15845543 10.1074/jbc.M503326200

[59] Tetzlaff, J.E., Putcha, P., Outeiro, T.F., Ivanov, A., Berezovska, O., Hyman, B.T. and McLean, P.J. (2008) CHIP targets toxic alpha-synuclein oligomers for degradation. J. Biol. Chem., 283, 17962–17968. 18436529 10.1074/jbc.M802283200 PMC2936239

[60] Kalia, L.V., Kalia, S.K., Chau, H., Lozano, A.M., Hyman, B.T. and McLean, P.J. (2011) Ubiquitinylation of alpha-synuclein by carboxyl terminus Hsp70-interacting protein (CHIP) is regulated by Bcl-2-associated athanogene 5 (BAG5). PLoS One, 6, e14695. 21358815 10.1371/journal.pone.0014695 PMC3040167

[61] Lowe, J., McDermott, H., Landon, M., Mayer, R.J. and Wilkinson, K.D. (1990) Ubiquitin carboxyl-terminal hydrolase (PGP 9.5) is selectively present in ubiquitinated inclusion bodies characteristic of human neurodegenerative diseases. J. Pathol., 161, 153–160. 2166150 10.1002/path.1711610210

[62] Yasuda, T., Nihira, T., Ren, Y.R., Cao, X.Q., Wada, K., Setsuie, R., Kabuta, T., Wada, K., Hattori, N., Mizuno, Y. et al. (2009) Effects of UCH-L1 on alpha-synuclein over-expression mouse model of Parkinson’s disease. J. Neurochem., 108, 932–944. 19141079 10.1111/j.1471-4159.2008.05827.x

[63] Cartier, A.E., Ubhi, K., Spencer, B., Vazquez-Roque, R.A., Kosberg, K.A., Fourgeaud, L., Kanayson, P., Patrick, C., Rockenstein, E., Patrick, G.N. et al. (2012) Differential effects of UCHL1 modulation on alpha-synuclein in PD-like models of alpha-synucleinopathy. PLoS One, 7, e34713. 22514658 10.1371/journal.pone.0034713 PMC3326048

[64] Kong, S.M., Chan, B.K., Park, J.S., Hill, K.J., Aitken, J.B., Cottle, L., Farghaian, H., Cole, A.R., Lay, P.A., Sue, C.M. et al. (2014) Parkinson’s disease -linked human PARK9/ATP13A2 maintains zinc homeostasis and promotes alpha-Synuclein externalization via exosomes. Hum. Mol. Genet., 23, 2816–2833. 24603074 10.1093/hmg/ddu099

[65] Tsunemi, T., Hamada, K. and Krainc, D. (2014) ATP13A2/PARK9 regulates secretion of exosomes and alpha-synuclein. J. Neurosci., 34, 15281–15287. 25392495 10.1523/JNEUROSCI.1629-14.2014 PMC4228131

[66] Li, S., Nie, K., Zhang, Q., Guo, M., Qiu, Y., Li, Y., Gao, Y. and Wang, L. (2019) Macrophage migration inhibitory factor mediates neuroprotective effects by regulating inflammation, apoptosis and autophagy in Parkinson's disease. Neuroscience, 416, 50–62. 31170483 10.1016/j.neuroscience.2019.05.052

[67] Raider, K., Ma, D., Harris, J.L., Fuentes, I., Rogers, R.S., Wheatley, J.L., Geiger, P.C., Yeh, H.W., Choi, I.Y., Brooks, W.M. et al. (2016) A high fat diet alters metabolic and bioenergetic function in the brain: a magnetic resonance spectroscopy study. Neurochem. Int., 97, 172–180. 27125544 10.1016/j.neuint.2016.04.008 PMC4900919

[68] Yamano, K., Matsuda, N. and Tanaka, K. (2016) The ubiquitin signal and autophagy: an orchestrated dance leading to mitochondrial degradation. EMBO Rep., 17, 300–316. 26882551 10.15252/embr.201541486 PMC4772979

[69] Garten, A., Schuster, S., Penke, M., Gorski, T., de Giorgis, T. and Kiess, W. (2015) Physiological and pathophysiological roles of NAMPT and NAD metabolism. Nat. Rev. Endocrinol., 11, 535–546. 26215259 10.1038/nrendo.2015.117

[70] Johnson, S. and Imai, S.I. (2018) NAD (+) biosynthesis, aging, and disease. F1000Res, 7, 132. 29744033 10.12688/f1000research.12120.1 PMC5795269

[71] Langston, J.W. (2017) The MPTP story. J. Parkinsons Dis., 7, S11–S19. 28282815 10.3233/JPD-179006 PMC5345642

[72] Quansah, E., Peelaerts, W., Langston, J.W., Simon, D.K., Colca, J. and Brundin, P. (2018) Targeting energy metabolism via the mitochondrial pyruvate carrier as a novel approach to attenuate neurodegeneration. Mol. Neurodegener., 13, 28. 29793507 10.1186/s13024-018-0260-x PMC5968614

[73] Yoshino, J., Baur, J.A. and Imai, S.I. (2018) NAD(+) intermediates: the biology and therapeutic potential of NMN and NR. Cell Metab., 27, 513–528. 29249689 10.1016/j.cmet.2017.11.002 PMC5842119

[74] Neumann, M., Kahle, P.J., Giasson, B.I., Ozmen, L., Borroni, E., Spooren, W., Muller, V., Odoy, S., Fujiwara, H., Hasegawa, M. et al. (2002) Misfolded proteinase K-resistant hyperphosphorylated alpha-synuclein in aged transgenic mice with locomotor deterioration and in human alpha-synucleinopathies. J. Clin. Invest., 110, 1429–1439. 12438441 10.1172/JCI15777 PMC151810

[75] Andrews, S. (2010) FastQC A quality control tool for high throughput sequence data. www.bioinformatics.babraham.ac.uk/projects/fastqc/, in press.

[76] Dobin, A., Davis, C.A., Schlesinger, F., Drenkow, J., Zaleski, C., Jha, S., Batut, P., Chaisson, M. and Gingeras, T.R. (2013) STAR: ultrafast universal RNA-seq aligner. Bioinformatics, 29, 15–21. 23104886 10.1093/bioinformatics/bts635 PMC3530905

[77] Love, M.I., Huber, W. and Anders, S. (2014) Moderated estimation of fold change and dispersion for RNA-seq data with DESeq2. Genome Biol., 15, 550. 25516281 10.1186/s13059-014-0550-8 PMC4302049

[78] Leek, J.T., Johnson, W.E., Parker, H.S., Jaffe, A.E. and Storey, J.D. (2012) The sva package for removing batch effects and other unwanted variation in high-throughput experiments. Bioinformatics, 28, 882–883. 22257669 10.1093/bioinformatics/bts034 PMC3307112

[79] Maze, I., Shen, L., Zhang, B., Garcia, B.A., Shao, N., Mitchell, A., Sun, H., Akbarian, S., Allis, C.D. and Nestler, E.J. (2014) Analytical tools and current challenges in the modern era of neuroepigenomics. Nat. Neurosci., 17, 1476–1490. 25349914 10.1038/nn.3816 PMC4262187

[80] Srinivasan, K., Friedman, B.A., Larson, J.L., Lauffer, B.E., Goldstein, L.D., Appling, L.L., Borneo, J., Poon, C., Ho, T., Cai, F. et al. (2016) Untangling the brain's neuroinflammatory and neurodegenerative transcriptional responses. Nat. Commun., 7, 11295. 27097852 10.1038/ncomms11295 PMC4844685

[81] Patro, R., Duggal, G., Love, M.I., Irizarry, R.A. and Kingsford, C. (2017) Salmon provides fast and bias-aware quantification of transcript expression. Nat. Methods, 14, 417–419. 28263959 10.1038/nmeth.4197 PMC5600148

[82] Love, M.I., Soneson, C., Hickey, P.F., Johnson, L.K., Pierce, N.T., Shepherd, L., Morgan, M. and Patro, R. (2020) Tximeta: reference sequence checksums for provenance identification in RNA-seq. PLoS Comput. Biol., 16, e1007664. 32097405 10.1371/journal.pcbi.1007664 PMC7059966

[83] Katz, Y., Wang, E.T., Airoldi, E.M. and Burge, C.B. (2010) Analysis and design of RNA sequencing experiments for identifying isoform regulation. Nat. Methods, 7, 1009–1015. 21057496 10.1038/nmeth.1528 PMC3037023

[84] Wang, E.T., Sandberg, R., Luo, S., Khrebtukova, I., Zhang, L., Mayr, C., Kingsmore, S.F., Schroth, G.P. and Burge, C.B. (2008) Alternative isoform regulation in human tissue transcriptomes. Nature, 456, 470–476. 18978772 10.1038/nature07509 PMC2593745

[85] Raudvere, U., Kolberg, L., Kuzmin, I., Arak, T., Adler, P., Peterson, H. and Vilo, J. (2019) g:Profiler: a web server for functional enrichment analysis and conversions of gene lists (2019 update). Nucleic Acids Res., 47, W191–W198. 31066453 10.1093/nar/gkz369 PMC6602461

[86] Shannon, P., Markiel, A., Ozier, O., Baliga, N.S., Wang, J.T., Ramage, D., Amin, N., Schwikowski, B. and Ideker, T. (2003) Cytoscape: a software environment for integrated models of biomolecular interaction networks. Genome Res., 13, 2498–2504. 14597658 10.1101/gr.1239303 PMC403769

[87] Wilcox, R.R. (2005) Introduction to Robust Estimation and Hypothesis Testing. Elsevier/Academic Press, Amsterdam, Boston.

[88] Langfelder, P., Zhang, B. and Horvath, S. (2008) Defining clusters from a hierarchical cluster tree: the dynamic tree cut package for R. Bioinformatics, 24, 719–720. 18024473 10.1093/bioinformatics/btm563

[89] Wetzels, R. and Wagenmakers, E.J. (2012) A default Bayesian hypothesis test for correlations and partial correlations. Psychon. Bull. Rev., 19, 1057–1064. 22798023 10.3758/s13423-012-0295-x PMC3505519

