# Supplementary material for: Failure of diet-induced transcriptional adaptations in alpha-synuclein transgenic mice
Source: Hum Mol Genet. 2022 Aug 24;32(3):450–61. doi: 10.1093/hmg/ddac205 (PMC9851747; doi:10.1093/hmg/ddac205)
Supplement: SuppFigures_ddac205 [file suppfigures_ddac205.pdf]

Supplementary Figure 1

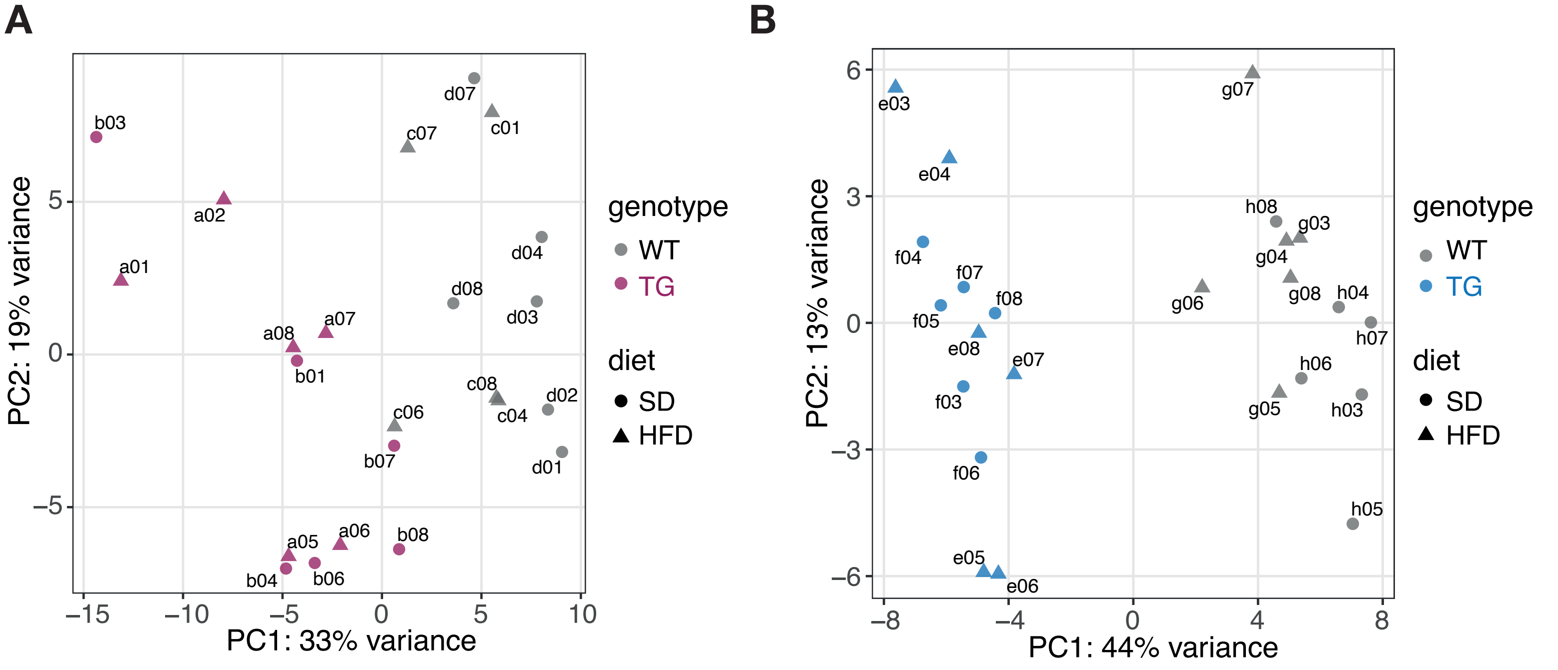

Supplementary Figure 1. Samples separate mainly according to genotype

(A) Principal component analysis of gene expression profiles for all brainstem samples. Axis percentages indicate variance explained by the first and second principle component.

(B) Analogous to (A) for hippocampal samples.

Supplementary Figure 2

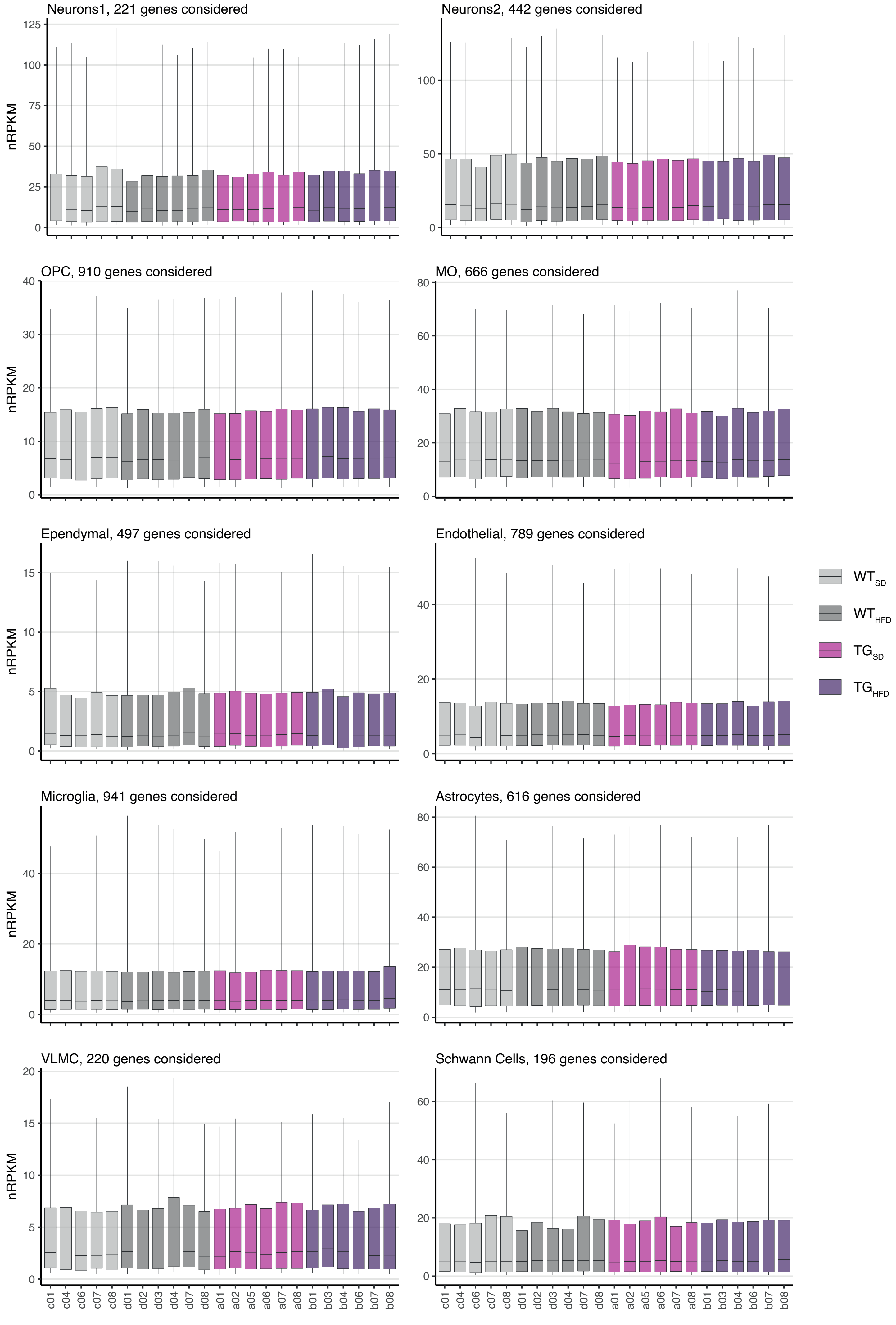

Supplementary Figure 2. Homogenous cell type-specific gene expression across brainstem samples

Cell type-specific gene expression per sample for ten reference cell types in brainstem [1]. Boxplots show geometric mean as well as 10th, 25th, 75th, and 90th quantile of expression values for each cell type. Number of considered genes per cell type above each plot.

Supplementary Figure 3

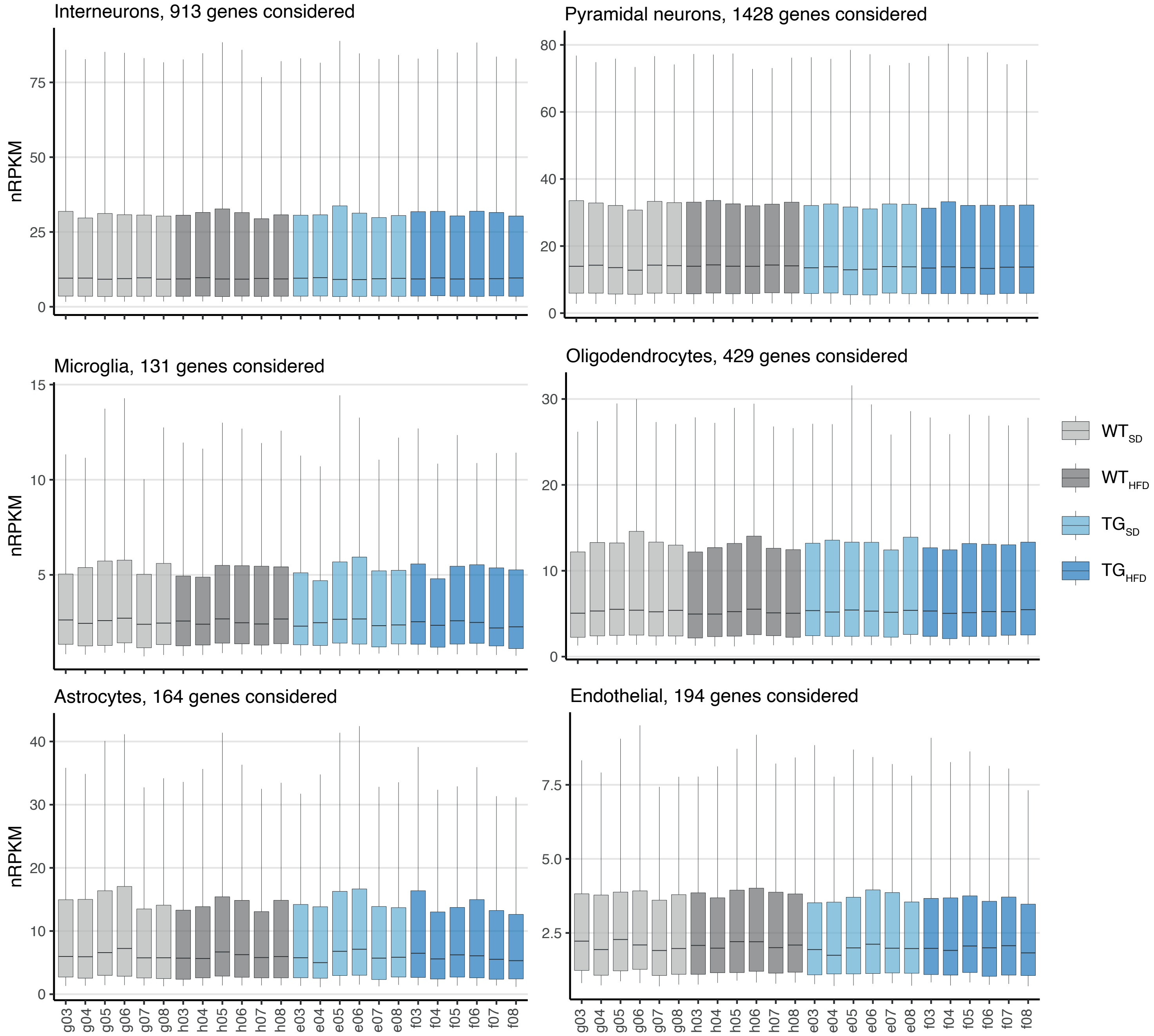

Supplementary Figure 3. Homogenous cell type-specific gene expression across hippocampal samples

Cell type-specific gene expression per sample for six main cell types identified in hippocampus [2]. Boxplots show geometric mean as well as 10th, 25th, 75th, and 90th quantile of expression values for each cell type. Number of considered genes per cell type above each plot.

Supplementary Figure 4

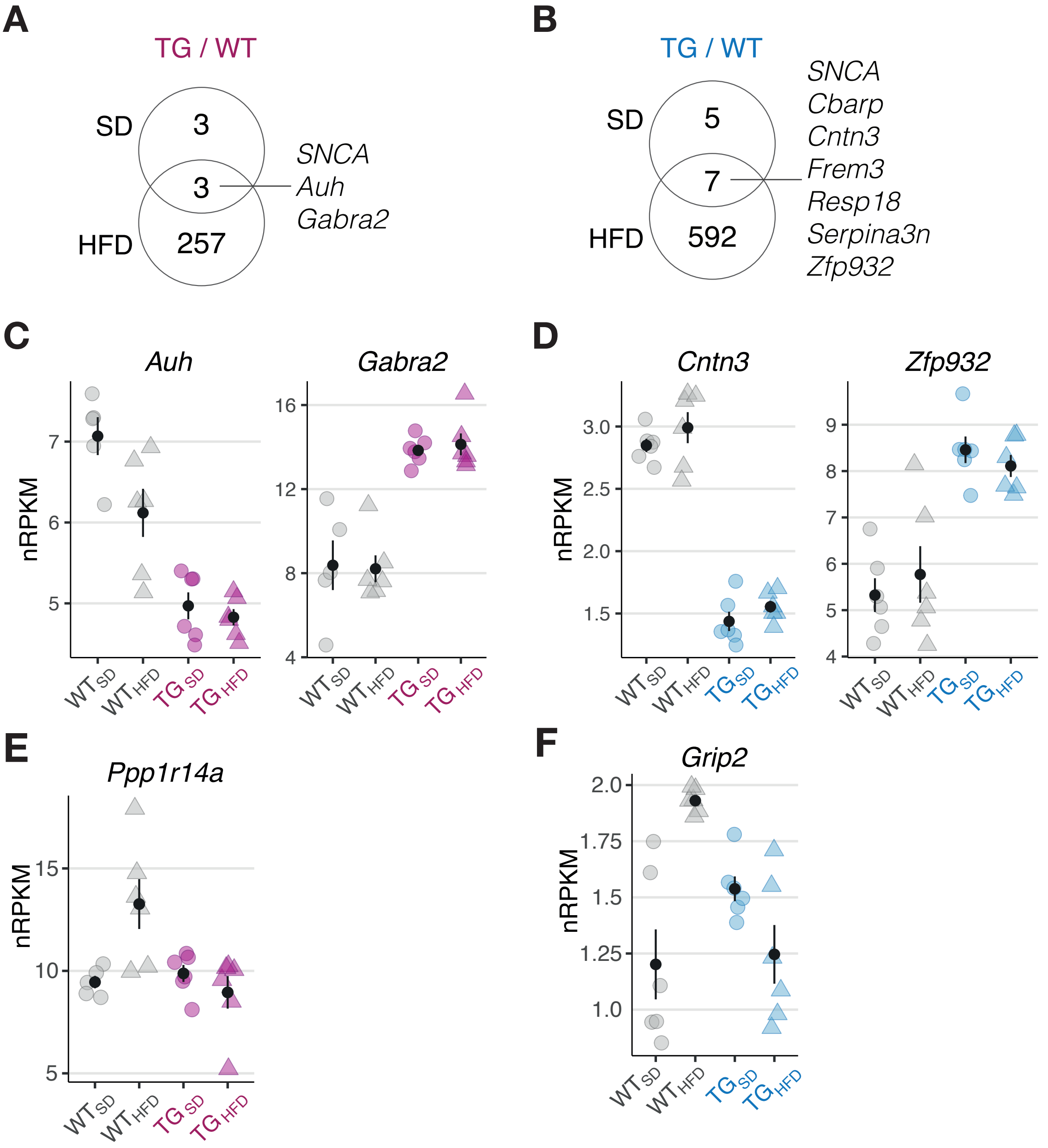

Supplementary Figure 4. Few genes show differential expression in TG mice independent of diet

(A) Venn diagram comparing DEGs identified in TG mice under standard and high fat diet in brainstem. Four common DEGs labeled.

(B) Analogous to (A) for DEGs in hippocampus. Eight shared genes labeled.

(C) Expression levels as normalized reads per kilobase per million (nRPKMs) for *Auh* and *Gabra2* across experimental groups in brainstem plotted as individual data points with mean ± SEM.

(D) Expression levels for *Cntn3* and *Zfp932* across experimental groups in hippocampus plotted as individual data points with mean ± SEM.

(E) Expression levels for *Ppp1r14a* identified with significant interaction between genotype and diet in brainstem plotted as individual data points with mean ± SEM.

(F) Expression levels for *Grip2* identified with significant interaction between genotype and diet in hippocampus plotted as individual data points with mean ± SEM.

Supplementary Figure 5

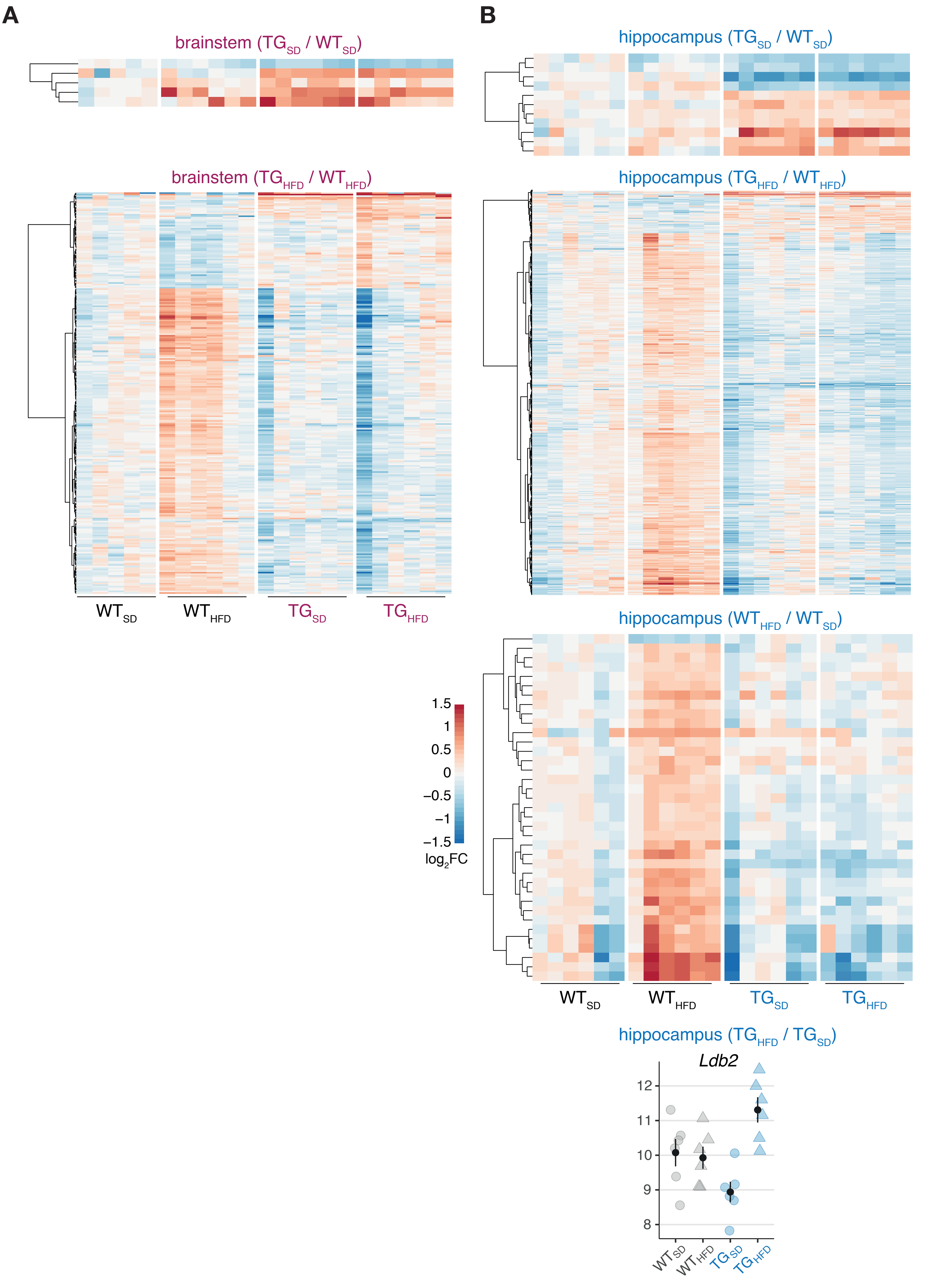

Supplementary Figure 5. Heatmaps for DEGs in each contrast

(A) Heatmap of hierarchically clustered expression profiles (*log_2* expression change relative to WT_{SD}) of DEGs for each contrast in brainstem across all experimental groups.

(B) Analogous to (A) for hippocampus, one additional DEG in TG_{HFD}/TG_{SD} plotted individually.

Supplementary Figure 6

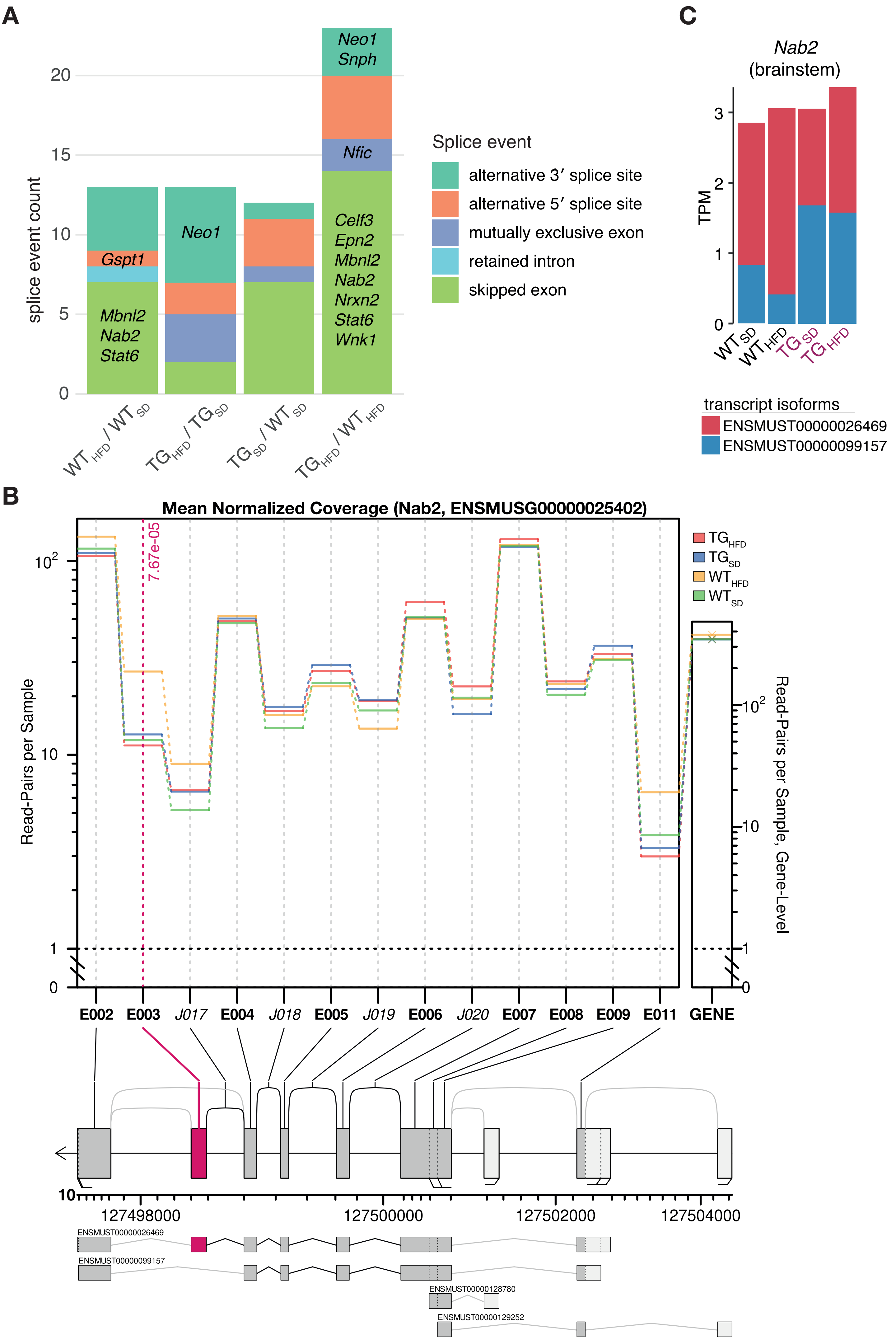

Supplementary Figure 6. MISO and JunctionSeq analyses identify genotype-diet dependent changes of splicing and transcript isoforms

(A) Number of splice events in hippocampal samples identified by *MISO*. Types of alternative splicing color-coded per primary contrast. Gene names shown for candidates also identified by *JunctionSeq*.

(B) Transcript isoform-specific expression levels of *Nab2* in brainstem samples across experimental groups. Plotted are mean transcripts per million (TPM) values per group obtained with *Salmon*.

(C) Mean normalized read coverage of all exonic and splice junction parts along the *Nab2* locus as per *JunctionSeq* analysis. Significantly altered entities highlighted. Lower part shows structure of all and affected transcript isoforms.

Supplementary Figure 7

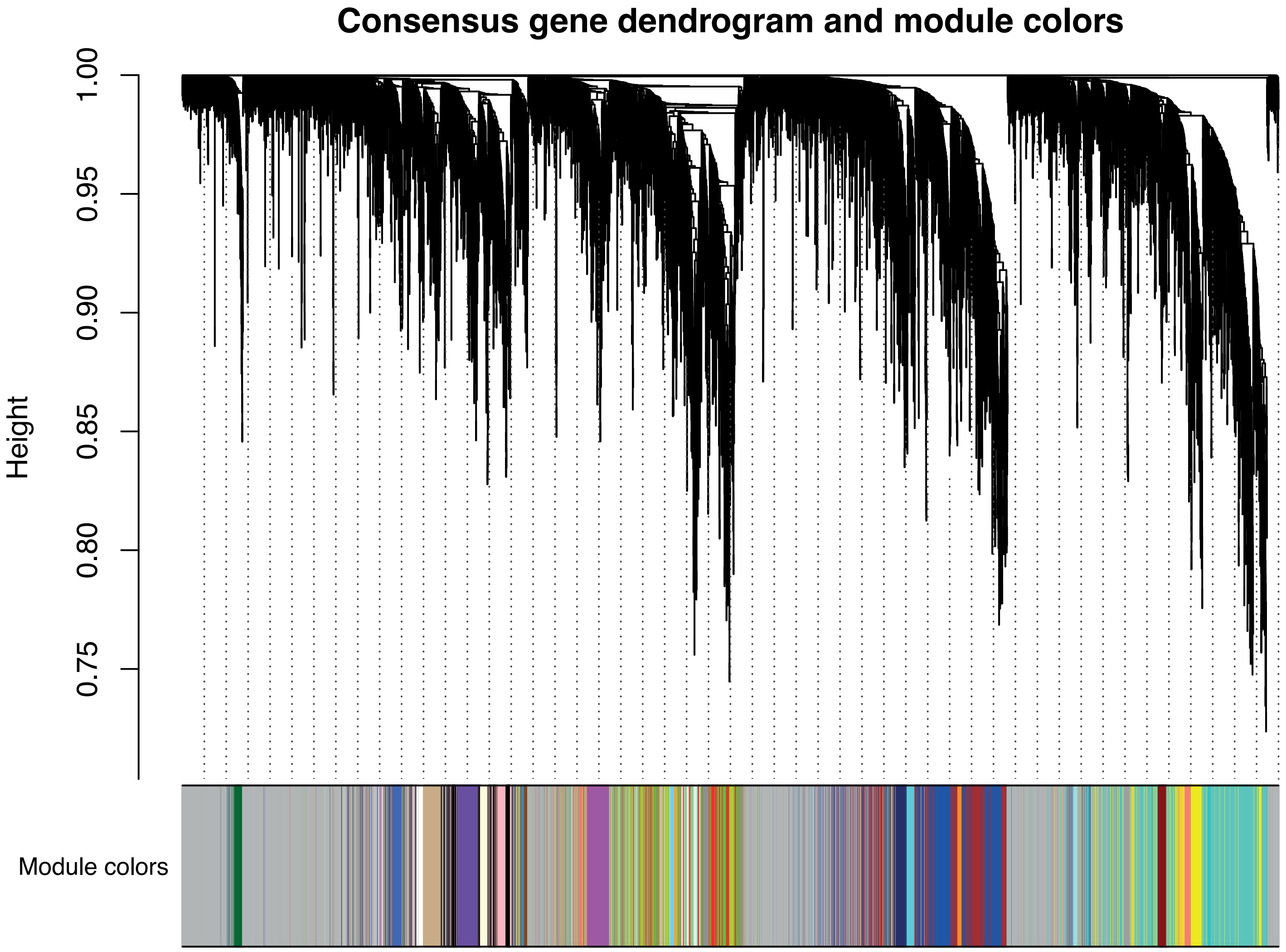

Supplementary Figure 7. Consensus WGCNA analysis identifies 32 modules

Representation of the hierarchical gene clustering tree leading to 32 modules of co-expressed genes. Leaves correspond to genes, and height reflects their closeness. Lower panel shows colors assigned to each module by the *Dynamic Tree Cut* and modules assigned consecutive to the *Merged Dynamic* method using a dissimilarity threshold of 0.1.

1. Liu, W., et al., *Single-cell RNA-seq analysis of the brainstem of mutant SOD1 mice reveals perturbed cell types and pathways of amyotrophic lateral sclerosis*. Neurobiol Dis, 2020. **141**: p. 104877.
2. Zeisel, A., et al., *Brain structure. Cell types in the mouse cortex and hippocampus revealed by single-cell RNA-seq*. Science, 2015. **347**(6226): p. 1138-42.
